# Insight into (Electro)magnetic Interactions within Facet-Engineered BaFe_12_O_19_/TiO_2_ Magnetic Photocatalysts

**DOI:** 10.1021/acsami.3c13380

**Published:** 2023-11-22

**Authors:** Szymon Dudziak, Cristina Gómez-Polo, Jakub Karczewski, Kostiantyn Nikiforow, Anna Zielińska-Jurek

**Affiliations:** † Department of Process Engineering and Chemical Technology, Gdansk University of Technology, G. Narutowicza Street 11/12, 80-233 Gdansk, Poland; ‡ Institute of Nanotechnology and Materials Engineering, Gdansk University of Technology, G. Narutowicza Street 11/12, 80-233 Gdansk, Poland; § Institute of Physical Chemistry, Polish Academy of Sciences, Kasprzaka Street 44/52, 01-224 Warsaw, Poland; ∥ Institute for Advanced Materials and Mathematics, INAMAT2, Public University of Navarre, Campus de Arrosadía, 31006 Pamplona, Pamplona, Spain

**Keywords:** BaFe_12_O_19_, TiO_2_, magnetic photocatalyst, magnetic field, crystal facets

## Abstract

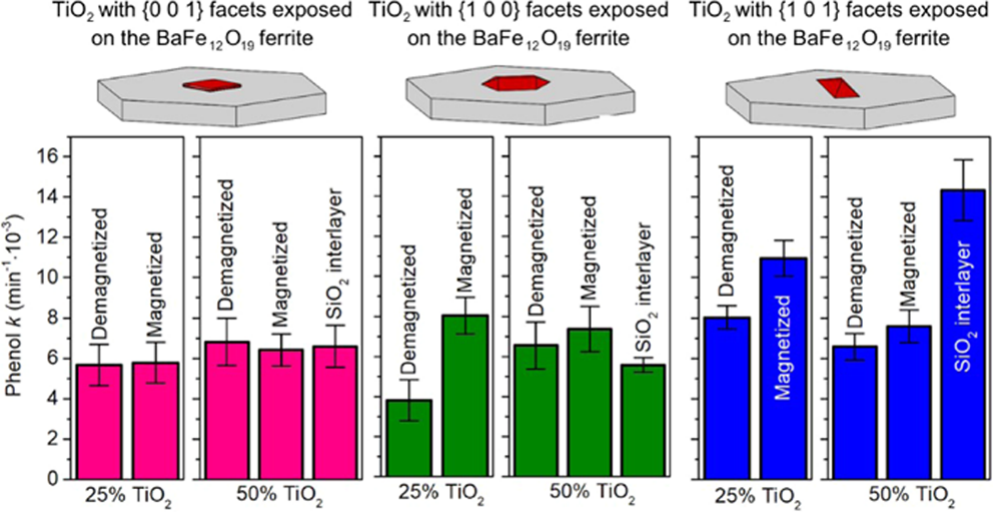

A series of facet-engineered TiO_2_/BaFe_12_O_19_ composites were synthesized through hydrothermal growth of both phases and subsequent deposition of the different, faceted TiO_2_ nanoparticles onto BaFe_12_O_19_ microplates. The well-defined geometry of the composite and uniaxial magnetic anisotropy of the ferrite allowed alternate interfaces between both phases and fixed the orientation between the TiO_2_ crystal structure and the remanent magnetic field within BaFe_12_O_19_. The morphology and crystal structure of the composites were confirmed by a combination of scanning electron microscopy (SEM) and X-ray diffraction (XRD) analyses together with the detailed study of BaFe_12_O_19_ electronic and magnetic properties. The photocatalytic activity and magnetic field effect were studied in the reaction of phenol degradation for TiO_2_/BaFe_12_O_19_ and composites of BaFe_12_O_19_ covered with a SiO_2_ protective layer and TiO_2_. The observed differences in phenol degradation are associated with electron transfer and the contribution of the magnetic field. All obtained magnetic composite materials can be easily separated in an external magnetic field, with efficiencies exceeding 95%, and recycled without significant loss of photocatalytic activity. The highest activity was observed for the composite of BaFe_12_O_19_ with TiO_2_ exposing {1 0 1} facets. However, to prevent electron transfer within the composite structure, this photocatalyst material was additionally coated with a protective SiO_2_ layer. Furthermore, TiO_2_ exposing {1 0 0} facets exhibited significant synergy with the BaFe_12_O_19_ magnetic field, leading to 2 times higher photocatalytic activity when ferrite was magnetized before the process. The photoluminescence emission study suggests that for this particular combination, the built-in magnetic field of the ferrite suppressed the recombination of the photogenerated charge carriers. Ultimately, possible effects of complex electro/magnetic interactions within the magnetic photocatalyst are shown and discussed for the first time, including the anisotropic properties of both phases.

## 1 Introduction

Photocatalysis presents a promising but challenging solution for various environmentally relevant processes, such as the generation of solar fuels and the purification of water and gas streams.^1−3^ However, large-scale application of heterogeneous photocatalysis still requires solving some challenges related to enhancing semiconductor activity and decreasing the recombination of photogenerated charge carriers participating in the redox reactions on the photocatalyst surface. In recent years, this has led to great attention focused on improving photocatalytic activity by designing and synthesizing different photocatalytic materials.^4−7^ More recently, studies have shown that photocatalytic processes might be optimized by alternating the exposition of the specific crystal planes.^8−10^ This is strictly connected with the anisotropy of the photocatalyst electronic structure, which depends on the direction inside the crystal structure, the specific arrangement of atoms at the interface, and the nature of the interface itself.^11−13^ Ultimately, the alternation of the photocatalyst morphology and exposed crystal facet is recognized as the state-of-the-art approach to optimize the performance in photocatalytic reactions. Another important technological issue in the application of such photocatalytic nanostructures is their separation after the reaction, which is especially important for water treatment processes. An interesting and promising strategy to overcome this challenge is to design a photocatalyst that can be separated in an external magnetic field. However, since the material that would be both ferromagnetic and highly photocatalytic active has not been found so far, this approach usually requires combining ferromagnetic/photocatalytic materials in the form of composites.^14−16^ While this approach seems generally straightforward, it creates a complex problem of possible interactions between both phases, which is especially important in the highlighted approach of photocatalyst shape engineering. Noteworthy, these interactions include not only possible electron transfer, which is a well-described phenomenon, but also possible interactions with the inner magnetic field that can be built-in within the ferromagnetic phase. At present, it is known that the introduction of the magnetic field can influence the photocatalytic activity and behavior of the photogenerated charge carriers.^17−19^ However, these studies generally do not include a possible anisotropy of the TiO_2_ electronic structure. In order to discuss such effects, it would be beneficial to achieve strict orientation between an external magnetic field and suspended particles, which is impossible due to the dispersed particles’ random orientation. As a consequence, at present, the possible effect of the magnetic field on TiO_2_ activity is limited only to general observations that are difficult to discuss with respect to the detailed photocatalyst structure. However, a well-defined orientation between two shape-engineered particles could lead to a fixed orientation between a built-in magnetic field and a combined photocatalyst. This possibility has inspired us to design a ferromagnet/photocatalyst composite with defined morphology to study both electronic and magnetic interactions that can be designed through their mutual shape engineering.

In this regard, the aim of the present study is to explain the following: (i) How changing the exposed crystal facets will influence the ferromagnet/photocatalyst interface and resulting performance? (ii) To what extent this interface can be optimized? (iii) Is the observed effect associated only with charge carrier transport or also with the built-in magnetic field? (iv) Does the magnetic/electronic interactions differ, depending on the system shape and orientation? To find answers to these questions, faceted anatase nanoparticles were prepared and used as the photocatalytic part of the composite due to their well-documented properties and performance.^20−22^ For the ferromagnetic phase, hexagonal BaFe_12_O_19_ was selected due to its high remanent magnetization, uniaxial magnetic anisotropy, relatively low Curie point, and preferred growth into two-dimensional (2D) microplates under hydrothermal conditions.^23,24^ Such a combination allows for the deposition of TiO_2_ with exposed {1 0 1}, {1 0 0}, and {0 0 1} crystal facets onto the same, preferentially exposed crystal facet of the ferrite, resulting in good control over both the interface and orientation between TiO_2_ and the vector of a built-in magnetic field, which is pointing at the specific direction, according to the uniaxial anisotropy. Furthermore, the relatively low Curie point of BaFe_12_O_19_ (about 450 °C) allows for demagnetization of the composite through calcination at temperatures that should not induce significant phase transformation of both materials. Finally, coating magnetic particles with a SiO_2_ layer to prevent electron transfer within the composite structure was also studied as a control to the samples in direct contact.

## 2 Methods

### 2.1 Synthesis of BaFe_12_O_19_ and BaFe_12_O_19_@SiO_2_

The synthesis of unmodified BaFe_12_O_19_ microplates was performed hydrothermally.^25^ Furthermore, purification of the NaOH solution from CO_3_^2–^ presence was performed based on the CaO-assisted procedure suggested by Sipos et al.^26^ Briefly, 100 cm^3^ of NaOH solution with a nominal concentration of 5 mol·dm^–3^ was mixed with 1 g of CaO, freshly calcined at 900 °C for 2 h, and ground to remove possible impurities. The mixture was sealed and left to react for 2 h under magnetic stirring, after which it was left for a few minutes to sediment most of the powder. Simultaneously, 4.5 g of Fe(NO_3_)_3_·9H_2_O and 0.4 g of Ba(NO_3_)_2_ were dissolved in 80 cm^3^ of deionized water with the addition of 0.5 g of poly(ethylene glycol) (PEG) inside the 200 cm^3^ lining of a hydrothermal reactor. The mixture was sealed with Al foil and magnetically stirred under a constant N_2_ flow for 20 min. After this time, a part of the sealing was opened, and 45 cm^3^ of the purified NaOH solution was immediately introduced to the mixture, passing through the two subsequent silica gel filters to help remove the last of the dispersed Ca particles. After precipitation, the magnetic stirrer and Al foils were removed, and the reactor was immediately closed and left to react at 250 °C for 24 h. After the reaction, the prepared powder was magnetically separated and washed with 10% (wt) CH_3_COOH and water. Finally, it was dried at 80 °C and calcined at 500 °C for 2 h before further characterization and modification.

Furthermore, surface coating of the prepared BaFe_12_O_19_ with SiO_2_ was achieved through a two-step condensation of tetraethyl orthosilicate (TEOS) in the presence of the ferrite particles. Specifically, 0.25 g of the ferrite was dispersed in the mixture of 100 cm^3^ of water with 5 cm^3^ of a 25% (wt) NH_4_OH solution, followed by the addition of 10 μL of TEOS under mechanical stirring. After 1 h of the initial reaction, magnetic particles were separated with a magnet. Further growth of the SiO_2_ layer was continued in the second dispersion using 20 cm^3^ of water and 100 cm^3^ of ethanol. During this step, two parts of 30 μL of TEOS were added with a 1 h interval and finally left to react for an additional 2 h. The final modified ferrite was separated with a magnet, washed with water, dried at 80 °C, and calcined at 500 °C for 2 h before further characterization and modification

### 2.2 Synthesis of Faceted TiO_2_

The synthesis of the faceted TiO_2_ particles, exposing the majority of the {0 0 1}, {1 0 0}, or {1 0 1} facets, was performed hydrothermally, following previously described procedures.^27−29^ Briefly, a mixture of 17 cm^3^ of titanium *tert*-butoxide, 30 cm^3^ of *n*-butanol, and 3.4 cm^3^ of a 50% (wt) HF solution was reacted at 210 °C for 18 h to achieve dominant exposition of the {0 0 1} facets. Furthermore, 1 g of commercial TiO_2_ P25 (Evonik) was treated either with 40 cm^3^ of a 10 mol·dm^–3^ NaOH solution at 120 °C for 20 h or with 40 cm^3^ of a 8.5 mol·dm^–3^ KOH solution at 200 °C for 16 h to obtain corresponding Na/K titanates as the precursors for the remaining samples. Dominant exposition of the {1 0 0} facets was achieved by reacting half of the obtained Na titanate with 100 cm^3^ of water at 210 °C for 24 h. For this reaction, the Na precursor was used directly after its formation without drying, only after washing it to a pH between 10 and 11. Finally, exposition of the {1 0 1} facets was achieved by washing the obtained K titanate precursor to the pH between 7 and 8, drying it at 80 °C and reacting 0.4 g of the dried powder with 100 cm^3^ of NH_4_OH/NH_4_Cl (0.3/0.3 mol·dm^–3^) buffer at 210 °C for 16 h.

All TiO_2_ samples were washed with water several times and dried at 80 °C prior to initial characterization and further combination with the ferrite. Control samples of pure TiO_2_ were also calcined at 500 °C for 2 h, similar to the case for the composites.

### 2.3 Preparation of the BaFe_12_O_19_/TiO_2_ and BaFe_12_O_19_@SiO_2_/TiO_2_ Composites

The preparation of magnetic composites of platelet BaFe_12_O_19_ combined with the prepared TiO_2_ with different morphologies was achieved based on attractive interactions between both suspended phases based on the performed ζ potential analyses. A typical procedure included individual dispersion of both materials in water under mechanical stirring and a further combination of the prepared suspensions at pH between 5 and 6. The final amount of the combined material was designed to be 0.1 g in each case and was suspended in a total volume of 200 cm^3^ of water, while specific amounts of BaFe_12_O_19_ and TiO_2_ varied. Furthermore, the pH of the suspension was controlled by using HCl and NH_4_OH. After stabilization, the prepared suspension was slowly evaporated under constant mechanical stirring, and the resulting powder was collected and calcined at 500 °C for 2 h. Modification with TiO_2_ of both unmodified BaFe_12_O_19_ and BaFe_12_O_19_@SiO_2_ was performed similarly.

### 2.4 Characterization of the Materials

The crystalline structure of the prepared materials was characterized using a Rigaku Mini Flex powder X-ray diffractometer (XRD) with a Cu Kα radiation source. The morphology and elemental composition of the samples were analyzed by using an FEI Quanta FEG 250 scanning electron microscope (SEM) combined with an Apollo-X SDD energy-dispersive X-ray spectroscopy detector (EDS). For the SEM/EDS analysis, samples were coated with a thin layer of Au to remove excess charge. Surface composition and oxidation states of the elements were analyzed based on the X-ray photoelectron spectroscopy (XPS) measurements using a PHI 5000 Versa Probe spectrometer with monochromatic Al Kα radiation. The high-resolution (HR) XPS spectra were collected with the hemispherical analyzer at a pass energy of 117.4 and an energy step size of 0.1 eV. Deconvolution of the spectra was performed using a Shirley background and a Gaussian peak shape with 30% Lorentzian character by using Casa XPS 2.3 software. Magnetic properties of the materials were analyzed using a Quantum Design MPMS XL7 SQUID magnetometer in the temperature range between 5 and 300 K. Absorption properties of the samples were determined using a Thermo Fisher Evolution 220 spectrophotometer for the wavelengths between 200 and 1100 nm, using BaSO_4_ as a diffusive reflectance standard (DR UV–vis), as well as a Nicolet Avatar 360 FTIR spectrometer for the wavenumbers between 400 and 4000 cm^–1^ (FTIR). For the FTIR measurements, approximately 0.5 mg of the sample was mixed with 200 mg of KBr and pressed into the pellet, which was further pretreated at 150 °C for 2 h under reduced pressure to minimize the amount of adsorbed water. The ζ potential of the samples was analyzed using Malvern Instruments Zetasizer 3000 apparatuses and HCl/NH_4_OH solutions for pH control. Electrochemical tests were performed using an Autolab PGSTAT204 potentiostat–galvanostat equipped with the FRA32 M module and using a 0.5 mol·dm^–3^ Na_2_SO_4_ solution as an electrolyte. Screen-printed electrodes were used during the measurements, with working and counter electrodes made of carbon and the reference electrode being Ag/AgCl. Prior to measurements, prepared samples were dispersed in a 1:1 vol. ethanol/water mixture and were drop-casted on the surface of the working electrode. After drying, the prepared layer was blocked with 2 μL of Nafion solution (1% in ethanol) and dried for final measurements. The Mott–Schottky analysis of the samples was performed based on the electrochemical impedance spectroscopy data, collected using 10 mV amplitude of the AC signal and 1000 Hz frequency. The space charge capacitance (*C*) was calculated using the relation *C* = −(2π·*f*·*Z*_im_)^−1^, where f is the frequency and *Z*_im_ is the imaginary part of the impedance.^30^ The flatband potential position was determined as the zero point of the fitted linear jump on the *C*^2–^ versus *E* graph. Photoluminescence spectra (PL) of the obtained samples were recorded using a Shimadzu RF-6000 spectrophotometer.

### 2.5 Computational Details

The electronic structure of the BaFe_12_O_19_ phase was simulated at the density functional theory (DFT) level using the projector augmented wave method (PAW) and Perdew–Burke–Ernzerhof (PBE) functionals with an energy cutoff of 500 eV, as implemented in the Quantum Espresso software package.^31−34^ During the calculations, the crystal structure was relaxed to the threshold of 10^–3^ Ry·Bohr^–1^ using 4 × 4 × 2 *k*-point sampling of the Brillouin zone. The simulation of the electronic structure was performed for the optimized geometry, including density of states (DOS) calculation for the 5 × 5 × 2 *k*-point grind and band structure calculation with 20 points between each high-symmetry point on the K-path.^35^ To get the best description of the electronic structure, calculations were repeated using different values of the Hubbard *U* parameter applied for Fe ions in order to take into account Coulombic interactions between different sites (*U*_Fe_ = 0, 3, 6, 9, 10, and 12 eV). The final structure is presented for the simulation that gave the best fit with the experimental results.

### 2.6 Photocatalytic Activity

The photocatalytic activity of the prepared materials was investigated in the reaction of phenol degradation. Typically, 25 mg of the sample was dispersed in the 25 cm^3^ of phenol solution with a concentration of 200 μmol·dm^–3^ inside the photoreactor, equipped with a quartz window. The prepared suspension was stirred mechanically and was aerated with a 4 dm·h^–1^ airflow for 30 min to achieve adsorption–desorption equilibrium. After this time, the reactor was irradiated with a 300 W Xe lamp located 60 cm from the reactor border. During the process, samples were collected before the stabilization, at the start of irradiation, and then every 5 min for a total of 30 min. Phenol concentration was estimated based on the conjugation reaction with 4-nitroaniline and colorimetric quantification for λ = 480 nm.

## 3 Results and Discussion

### 3.1 Structure and Morphology of the Prepared BaFe_12_O_19_ and BaFe_12_O_19_@SiO_2_

**Figure 1:**
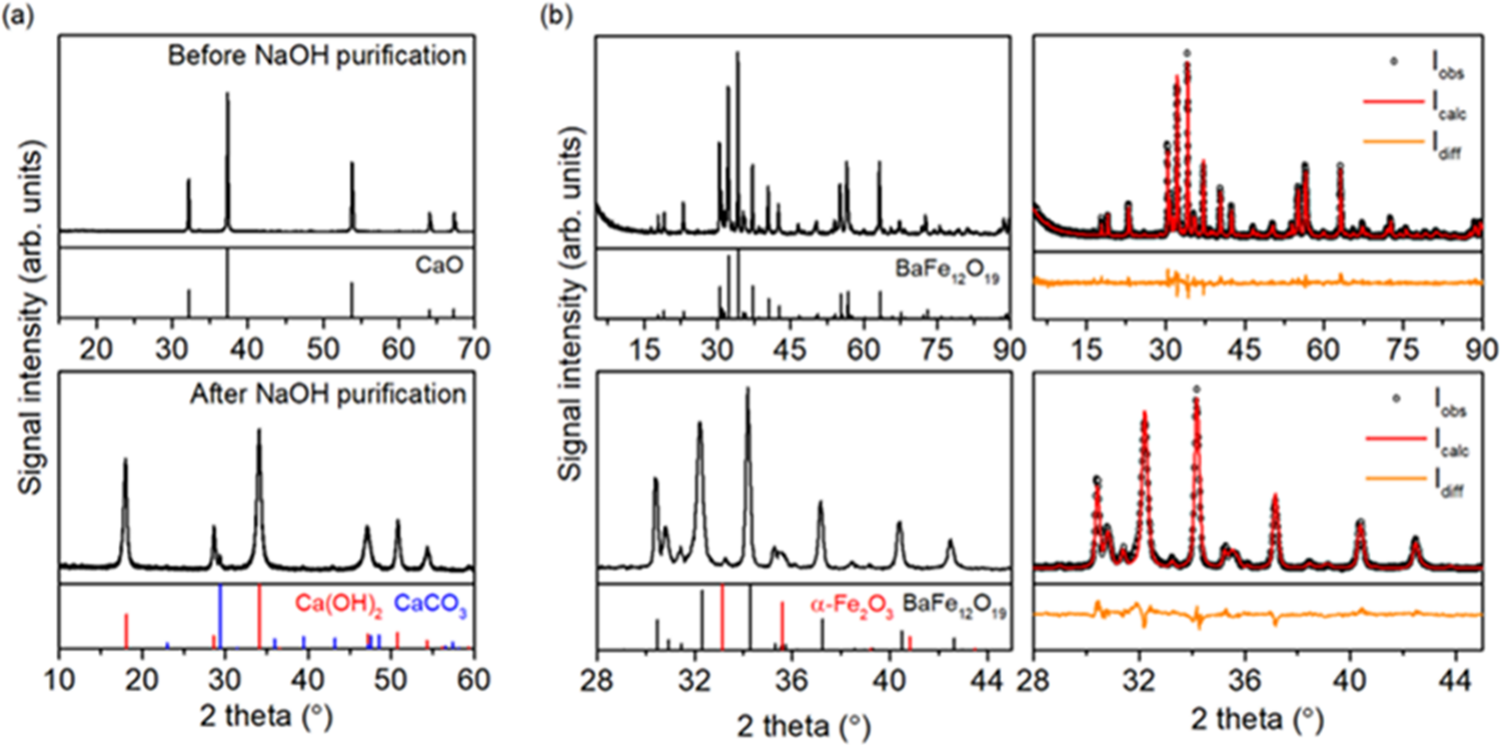
X-ray diffraction patterns of (a) CaO used for purification of the NaOH solution before and after the reaction and (b) final obtained BaFe_12_O_19_ sample after calcination at 500 °C for 2 h. Panel (b) shows patterns itself and Rietveld-refined profiles with calculated residuals.

Preparation of single-phase BaFe_12_O_19_ usually requires either high calcination temperatures or a high excess of Ba introduced to the hydrothermal reaction. This is mostly associated with the problem of the Ba^2+^ reaction with CO_2_/CO_3_^2–^, which results in the formation of BaCO_3_, leading to limited incorporation of barium into the hexaferrite structure and simultaneous cocreation of hematite α-Fe_2_O_3_. The further reaction between BaCO_3_ and α-Fe_2_O_3_ requires at least 600 °C to complete the formation of BaFe_12_O_19_.^36^ However, temperatures exceeding 800 °C are commonly needed to obtain single-phase ferrite.^37^ Focusing on the possible application of BaFe_12_O_19_ in photocatalytic processes, calcination at such temperatures is not desired, as it can lead to significant aggregation and sintering of the particles, reducing their surface area and limiting control over their further modification. On the other hand, the hydrothermal approach in Ba-rich solutions generally leads to the formation of very elongated BaFe_12_O_19_ microplates with visibly decreased coercivity, magnetic saturation, and remanence.^38^ As this study aims to analyze the potential impact of the BaFe_12_O_19_ inner magnetic field on the photocatalytic activity, softening its properties is also not desired. In this regard, it is necessary to eliminate CO_2_/CO_3_^2–^ from the synthesis by purifying NaOH solution from Na_2_CO_3_ presence through the reaction with freshly calcined CaO, as suggested by Sipos et al.^26^ As shown in Figure 1a, this process resulted in precipitation of about 3.6% (wt) calcite CaCO_3_, showing successful solution purification. However, most CaO was simultaneously transformed to Ca(OH)_2_, affecting the actual concentration of the OH^–^ ions introduced to the synthesis and influencing BaFe_12_O_19_ nucleation. In this regard, in the present study, the optimal amount of the purified NaOH was determined experimentally to be around 45 cm^3^, while especially lower amounts resulted in the clear presence of α-Fe_2_O_3_ (>5% wt). Ultimately, BaFe_12_O_19_ prepared under such conditions and further calcined at 500 °C for 2 h showed high purity, as presented in Figure 1b, with the presence of α-Fe_2_O_3_ reduced to 1.8% (wt), as determined through Rietveld refinement of the whole pattern (χ^2^ = 1.53). This amount of α-Fe_2_O_3_ resulted from capturing atmospheric CO_2_ during the precipitation, which is likely impossible to avoid completely. Nevertheless, the amount of α-Fe_2_O_3_ is very small and will not influence, e.g., the overall magnetic properties, as shown later. Modification of obtained BaFe_12_O_19_ with SiO_2_ resulted in no change in its diffraction pattern, which is in accordance with the expected amorphous nature of the obtained silica particles.

**Figure 2:**
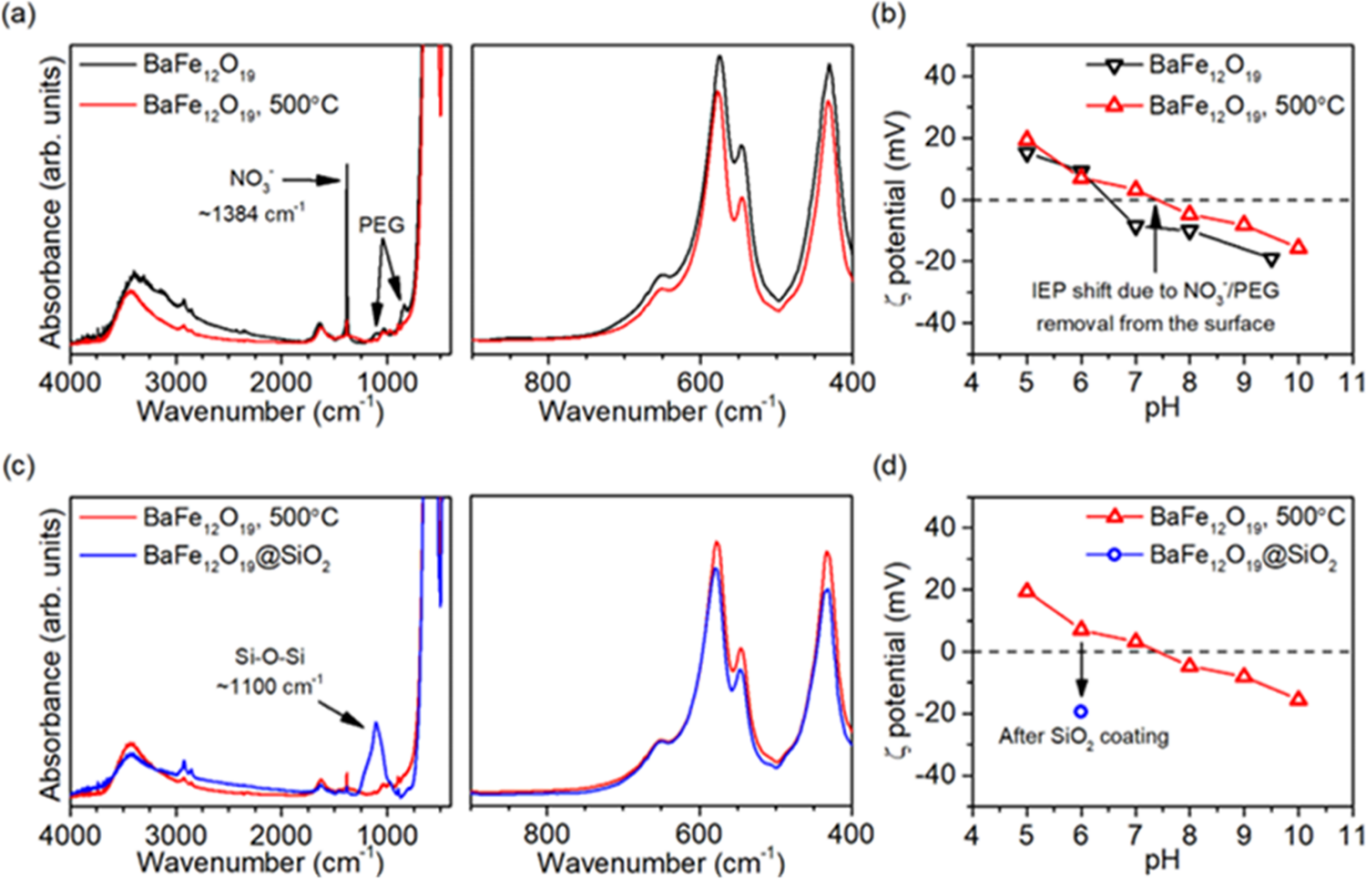
Comparison of the FTIR spectra (a, c) and ζ potential change with pH (b, d) of the prepared BaFe_12_O_19_ samples directly after the synthesis and calcination at 500 °C for 2 h and after modification with SiO_2_.

Furthermore, calcination of the synthesized BaFe_12_O_19_ was necessary to purify its surface from residual NO_3_^–^ and PEG molecules that were left after the synthesis. This was controlled by a combination of FTIR and zeta (ζ) potential measurements, as shown in Figure 2. The NO_3_^–^ presence after the synthesis is observed as a sharp peak at ∼1384 cm^–1^,^39^ and the residual PEG molecules are noticed due to weak bands at 840–860 and ∼1100 cm^–1^, characteristic for C–O and C–C vibrations, respectively, observed previously for PEG.^40^ Both of these signals are mostly hindered after the calcination, which is accompanied by the shift of the observed isoelectric point (IEP) to the higher pH value, similar to the one reported previously for the BaFe_12_O_19_ prepared by calcination at 1000 °C.^41^ Both of these observations prove successful purification of the ferrite surface before further investigation.

Moreover, after coating with SiO_2_, a broad, asymmetric signal centered at 1100 cm^–1^ appeared, which is in very good agreement with the known signals for the analogical silica structures.^42,43^ A simultaneous shift of the measured ζ potential at a pH of 6 to a strongly negative value further proves successful modification with SiO_2_. During both described procedures, no significant changes in the absorbance characteristic for metal oxides (<700 cm^–1^) are noticed, suggesting that their effect on the structure of BaFe_12_O_19_ itself is limited.

**Figure 3:**
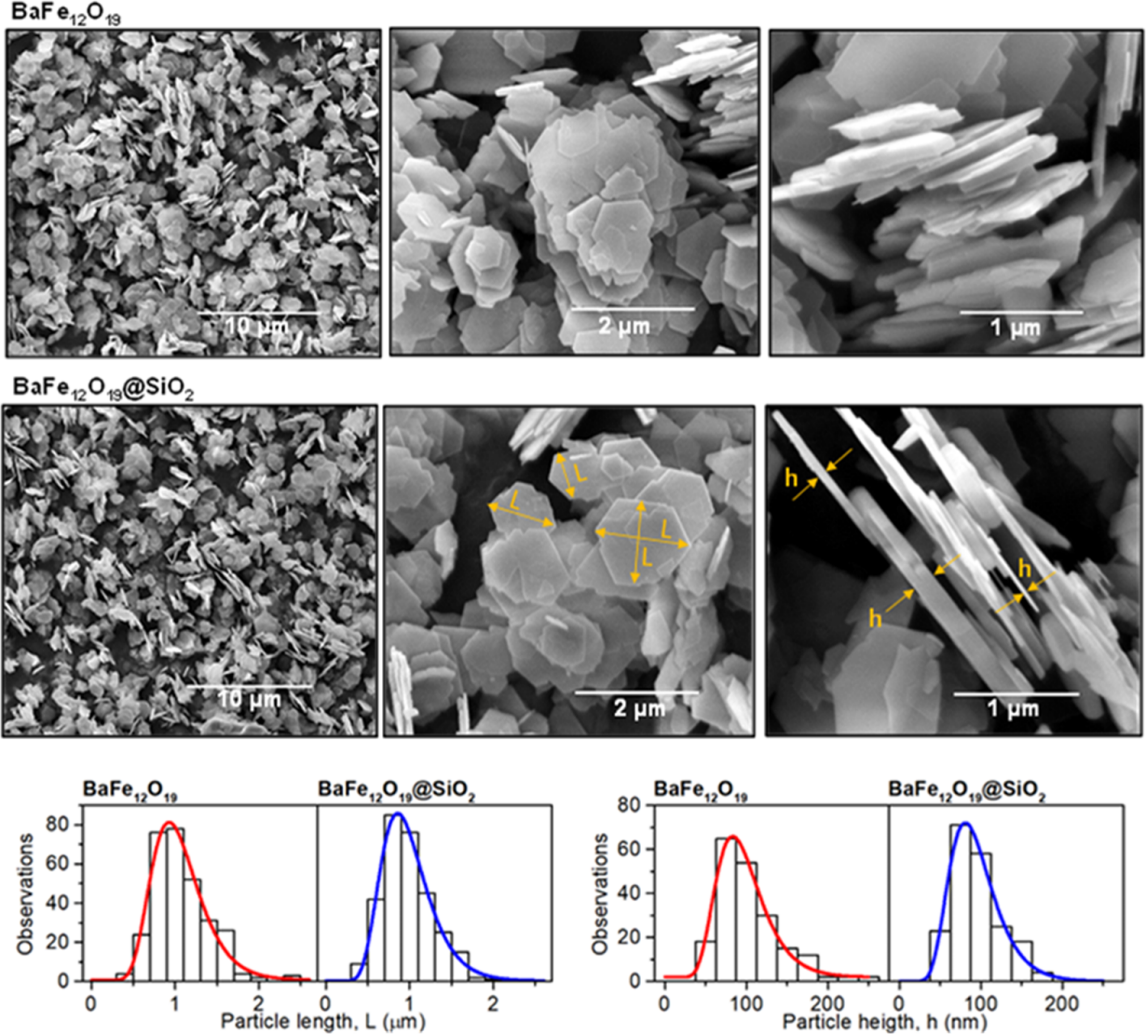
SEM images of the final calcined BaFe_12_O_19_ sample, before and after modification with SiO_2_, together with the statistical distribution of the observed dimensions *L* (300 counts per sample) and *h* (200 counts). Exemplary dimensions are highlighted for the BaFe_12_O_19_@SiO_2_ sample.

The morphology of the final calcined BaFe_12_O_19_ sample was observed in detail before and after modification with SiO_2_. As shown in Figure 3, almost no difference was visible between both materials, and hexagonal-shaped microplates with clean facets were systematically observed in all images. Based on the statistical distribution of the observed dimensions, the mean length and height of the plates were about 1000 mm × 95 nm. Detailed values are shown in Table 1, with errors presented as standard deviations of the fitted log-normal distributions. Such morphology is in agreement with previous studies on the preparation of BaFe_12_O_19_, which shows that large facets are oriented perpendicularly to the [0 0 1] vector of the ferrite crystal structure.^38^ Although the atomic geometries of these facets are not known at the moment, it is important to notice that they are also perpendicular to the easy magnetization axis of BaFe_12_O_19_,^23^ and therefore, the vector of ferrite inner magnetic field will tend to point at the exposed surface.

**Table 1:** Mean Observed Dimensions of the BaFe_12_O_19_ Plates and Summation of the Elemental Analysis

sample	particle length (μm)	particle height (nm)	particle volume^a^ (μm^3^)	EDS Fe/Ba (at.)	EDS Si (atom %)	XPS Si (atom %)
BaFe_12_O_19_	1.06 ± 0.32	97 ± 32	9.441 × 10^–2^	11.0 ± 0.6	n.d.	n.d.
BaFe_12_O_19_@SiO_2_	0.99 ± 0.31	95 ± 31	7.971 × 10^–2^	10.9 ± 0.6	n.d.	8.1

a Calculated assuming the length of the particle as double apothem of the regular hexagon.

Furthermore, the consistency of the observed morphology and exposition of the clean facets in both cases suggests that SiO_2_ forms a very thin layer on the surface without significant aggregation into bigger entities. This was further confirmed with the EDS analysis, performed over approximately 65 μm^2^ of the sample, which showed no Si enrichment for the SiO_2_-coated sample. As the EDS analysis has low sensitivity for the detection of surface species, this suggests that Si–O–Si structures observed in the FTIR spectrum are predominantly present on the surface.

**Figure 4:**
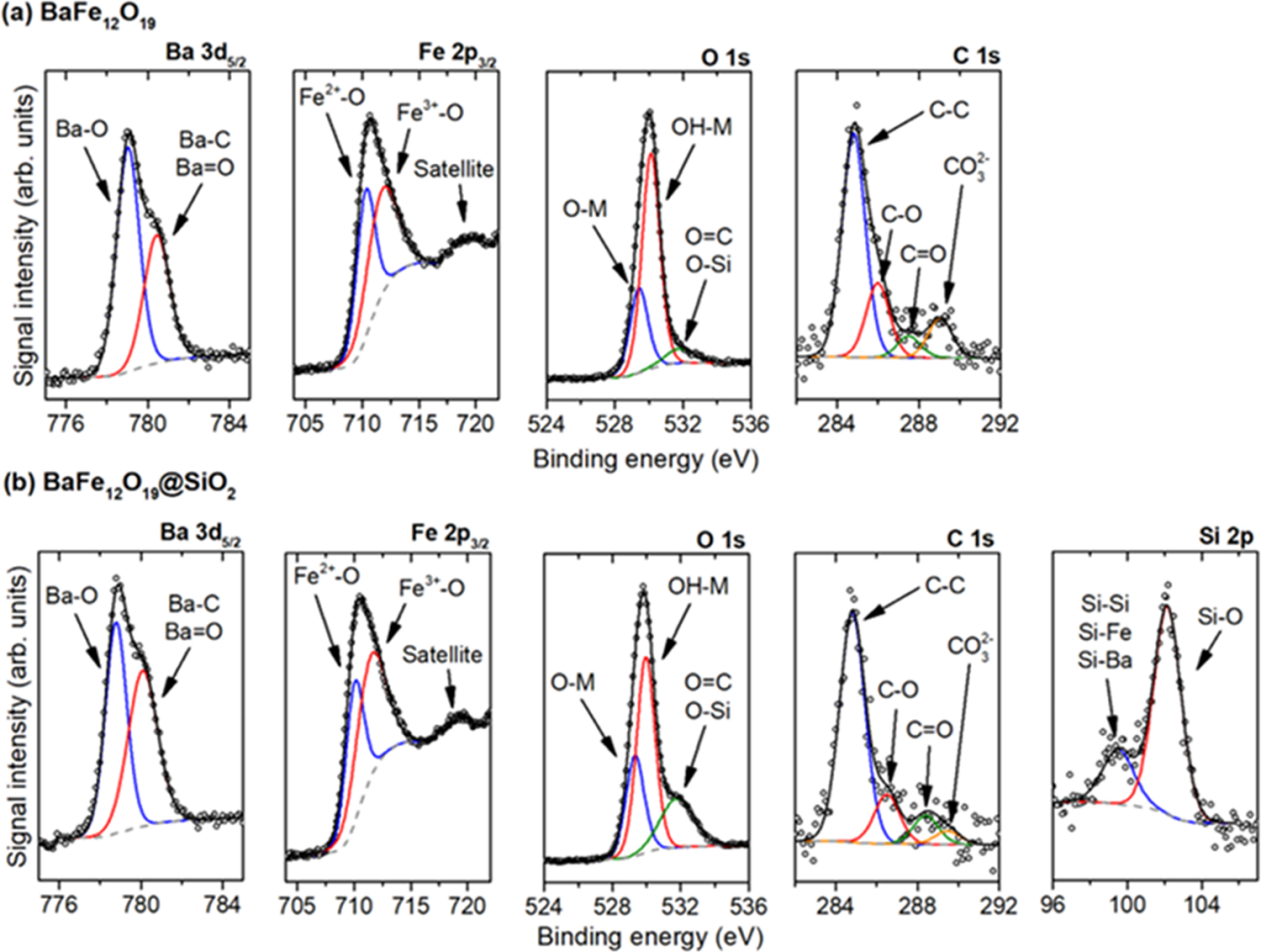
Deconvolution of the observed XPS signals for both unmodified (a) and SiO_2_-modified (b) BaFe_12_O_19_ samples. Deconvolution of the Ba 3d_5/2_ peak is presented after the extraction of the Fe_LMM_ signal.

Finally, the detailed surface composition of both samples was analyzed based on the XPS results, as shown in Figure 4. For the BaFe_12_O_19_@SiO_2_ sample, the presence of 8.1% (at.) silicon was observed, ultimately proving its surface presence. Deconvolution of the Si peak in Figure 4b has shown that Si exists mostly as the nonstoichiometric oxide, which is further confirmed with the increased O 1s signal for the binding energy of ∼532 eV. Noteworthy, some of the Si exist in the metallic form, originating from the Si–Si bonds within the SiO_*x*_ structure or Si–Ba/Si–Fe that could be formed between the ferrite surface and the growing SiO_2_ layer. Assuming that the silica layer forms a very thin structure, which is in accordance with its presence not observed during the SEM/EDS studies, the appearance of a relatively strong signal originating from the Fe/Ba–Si bonds formed at the interface seems reasonable. No presence of Si was observed in the case of the unmodified sample.

Besides, both materials are fairly similar, showing no significant differences. In both cases, surface enrichment with barium is observed, connected to the existence of two different Ba states. Especially, the second state at higher binding energy (780.5 eV) is not related to the BaFe_12_O_19_ structure^44−46^ and shows BaCO_3_ presence. This is especially visible for the unmodified sample, clearly confirmed with the C 1s signal at ∼289 eV and the O 1s signal at ∼532 eV that give an almost stoichiometric ratio of all elements (calculated Ba/C/O for these states is 1:0.92:3, after correction for the C=O bonds with the C 1s signal at ∼287.5 eV). As already mentioned, the formation of BaCO_3_ results probably from the capture of atmospheric CO_2_ during precipitation with NaOH, despite its previous purification, and is in agreement with the trace amount of α-Fe_2_O_3_ observed in the XRD pattern. Nevertheless, the absolute presence of Ba species associated with the carbonate is only ∼1.3% (at.), showing that the vast majority of the sample surface results strictly from the BaFe_12_O_19_ structure. Moreover, for both samples, a detailed analysis of the Fe 2p_3/2_ signal suggests the existence of two different Fe states with lower and higher oxidations in the oxide structure. However, this is probably the effect of different Fe^3+^ coordination inside the BaFe_12_O_19_ crystal lattice (octahedral, tetrahedral, and bipyramidal Fe^3+^ sites are present in the bulk structure^14^), which are known to affect Fe 2p_3/2_ peak shape.^47^ In this regard, the dominant presence of Fe^3+^ is confirmed with a satellite peak separated from the main Fe 2p_3/2_ signal, which is not observed for the Fe^2+^ ions.^48^ Therefore, the Fe signal is in agreement with the expected BaFe_12_O_19_ structure. For the SiO_2_-modified sample, a slight increase in the Ba and Fe states with higher binding energy results probably from the additional calcination of the sample, forming more oxidized species at the surface.

### 3.2 Magnetic and Electronic Properties of the Prepared BaFe_12_O_19_ and BaFe_12_O_19_@SiO_2_

**Figure 5:**
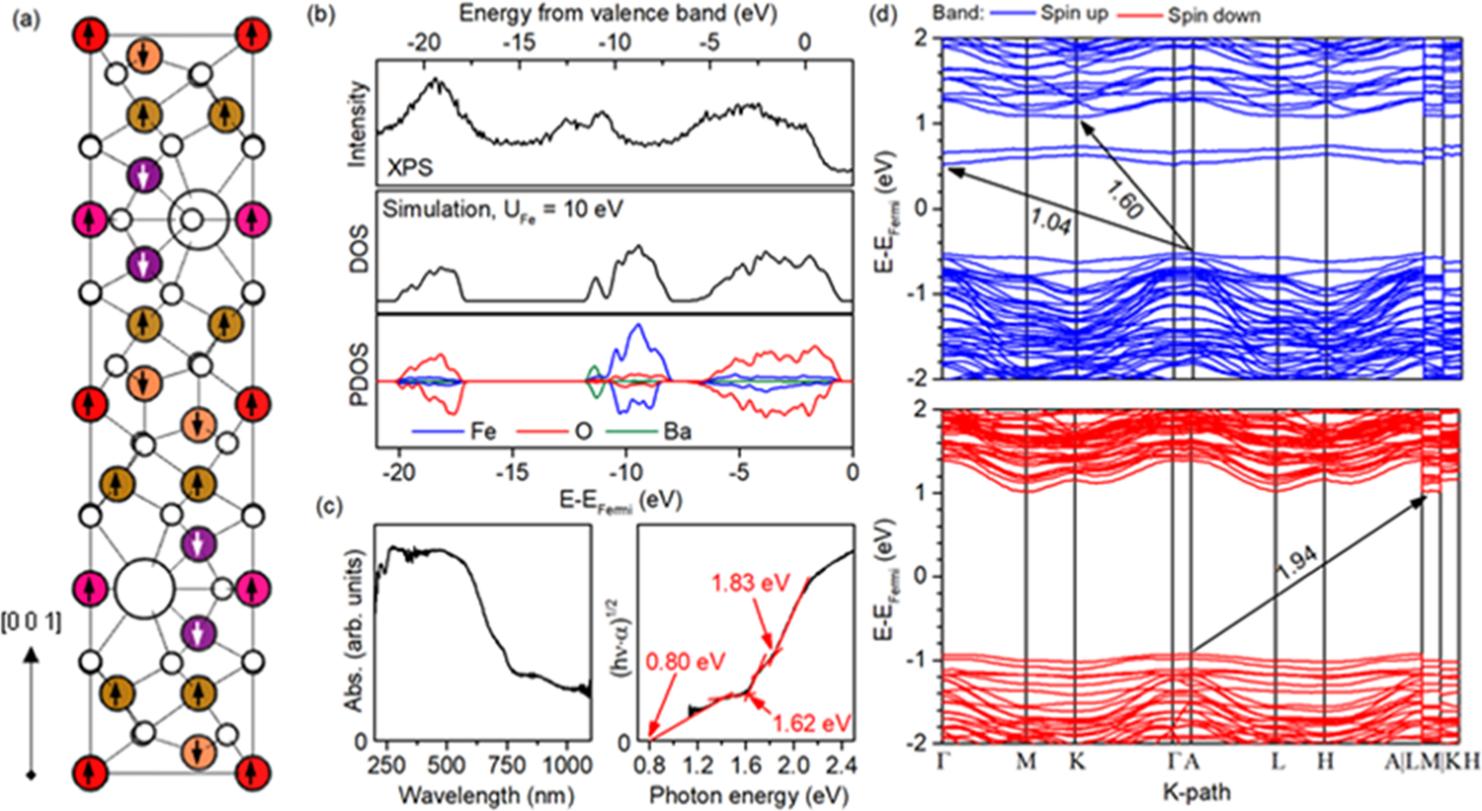
Summation of the BaFe_12_O_19_ electronic structure with a minimum-energy spin configuration of the Fe atoms, as shown in panel (a), where Fe sites with the same coordination are marked with the same color (big white spheres are Ba and small white spheres are O atoms). The simulated density of states distribution, compared to the observed density of occupied states below valence band top (b), band gap determination from absorbance results (c), and detailed band structure (d).

Due to the relatively low amount of information about BaFe_12_O_19_ application in the photocatalysis process, its electronic structure was investigated in detail before combining it with TiO_2_. It was started by simulating the density of state distribution and band structure, assuming minimum-energy spin configuration between the subsequent Fe layers,^49^ as shown in Figure 5a. During these studies, different values of the Hubbard parameter *U* were applied for the Fe atoms to achieve a possibly good correlation between the simulated structure and experimental results obtained from the XPS and DR/UV–vis analyses (*U*_Fe_ = 0, 3, 6, 9, 10, and 12 eV). Ultimately, the best agreement was obtained for *U*_Fe_ = 10 eV. Specifically, as shown in Figure 5b, the simulated band structure down to *E* – *E*_Fermi_ = −21 eV is in good agreement with the XPS results, showing three subsequent bands, in accordance with, e.g., a previous study by Atuchin et al.^46^ Interestingly, the detailed band gap analysis presented in Figure 5c suggests the existence of up to three electron transitions, which was further supported by band structure calculations (Figure 5d). The lowest energy transition occurs between the *A*/Γ symmetry points in the spin-up component, with a computed energy of about 1 eV. Interestingly, this is in agreement with the absorbance studies, which show visible absorption down to the full measurement range of the apparatus (∼1.1 eV). Extrapolation of this trend beyond experimental data suggests that the minimum excitation energy can be as low as 0.80 eV, which is a bit lower than a value obtained from the simulation. However, it should be pointed out that in this case, a detailed comparison of these values is difficult due to the various factors present in both measurements (arbitrary applied *U* value during the simulation, which affected the exact position of the band, the possible presence of some defect states in the analyzed sample, and extrapolation of the experimental value). Nevertheless, both absorbance and DFT studies agree that this low-energy transition can be distinguished for BaFe_12_O_19_, which might affect its photocatalytic performance. Furthermore, the second signal observed in the absorbance spectra, with a minimum energy of 1.62 eV, belongs to the indirect excitation between the A and K high-symmetry points, also in the spin-up part of the structure. As shown, the analogous transition in the spin-down component is associated with a bit higher energy (1.94 eV from simulations, between the A and minimum at the L → M path), in accordance with the absorption spectra (third transition at 1.83 eV). However, it should be noted that above ∼1.8 eV, direct excitation of the electrons with the up-spin might also occur, which will cause overlapping of these signals.

**Figure 6:**
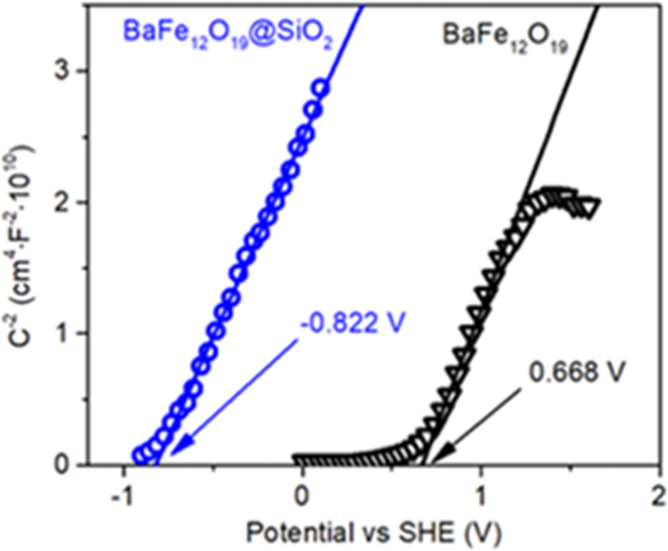
Mott–Schottky plot and determined flatband potentials for the BaFe_12_O_19_ and BaFe_12_O_19_@SiO_2_ samples.

Furthermore, band structure studies were followed with Mott–Schottky analysis to determine the ferrite position of the conduction band edge (CB) based on the determined flatband potential. As shown in Figure 6, the conduction band edge for BaFe_12_O_19_ is estimated at 0.688 V relative to the standard hydrogen electrode. This value is in good agreement with the CB position of, e.g., α-Fe_2_O_3_, being approximately 0.6–0.8 V, as reported in the literature.^50,51^ Noteworthy, when BaFe_12_O_19_ was modified with the SiO_2_ layer, the effective CB edge was shifted to the significantly lower potential of −0.822 V. While it is unlikely that this value represents the conduction band of SiO_2_ itself and is probably influenced by both silica nonstoichiometry and creation of the interface between SiO_2_ and other surface species observed during XPS studies, it does show that effective potential of the electrons present at the BaFe_12_O_19_@SiO_2_ surface is significantly lowered. Above all, this will be used to hinder electron transfer from TiO_2_ to BaFe_12_O_19_, as shown in the further sections of the present study.

**Figure 7:**
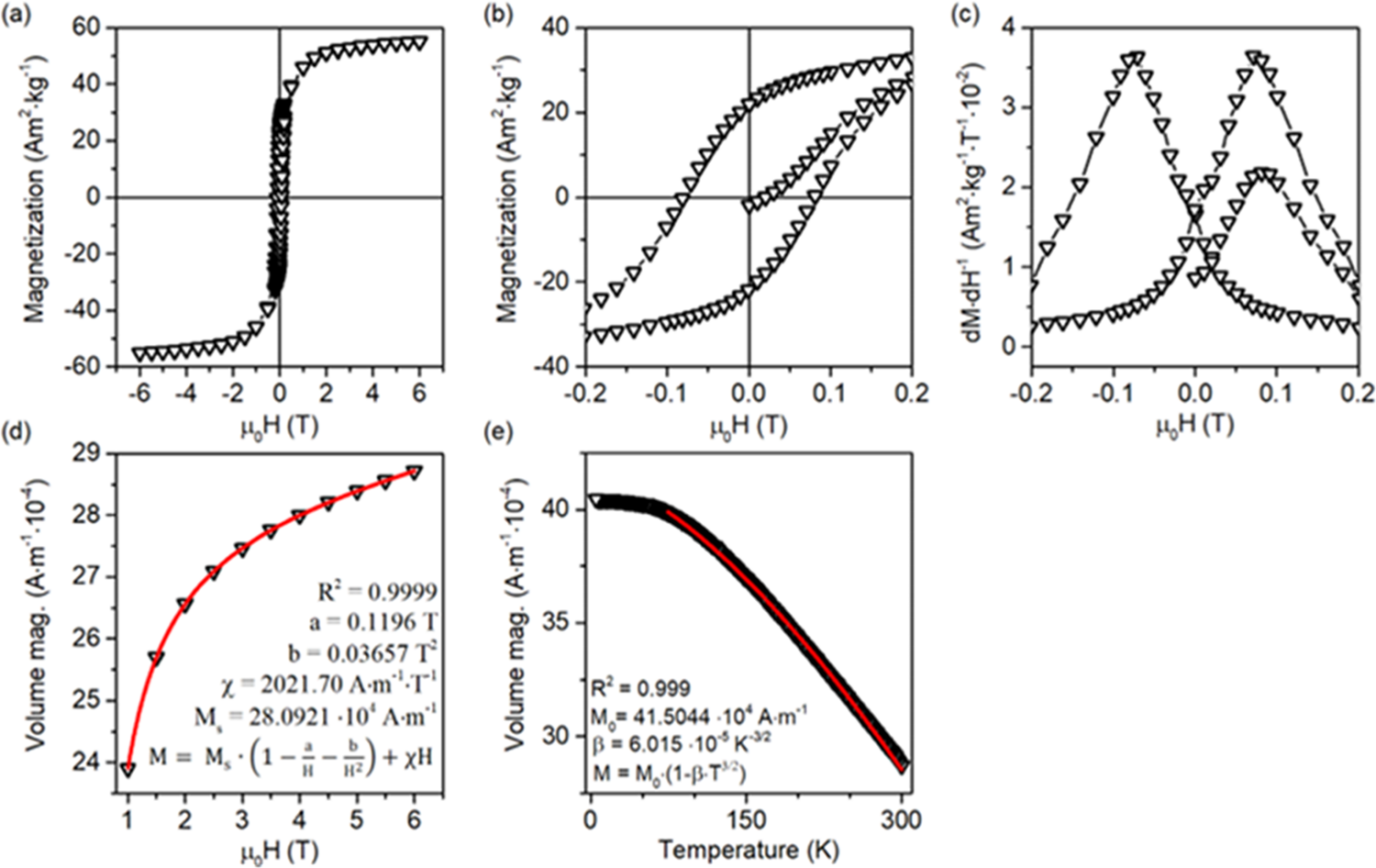
Summation of the magnetization studies for the prepared BaFe_12_O_19_ sample: (a, b) magnetization hysteresis with (c) calculated d*M*/d*H* derivative, (d) results of the high-field magnetization fitting to the LAS equation, and (e) high-field *M* versus T data.

The analysis of the electronic properties was followed with magnetization studies, and the results are summarized in Figure 7. Depending on the analysis details, the obtained mass magnetization (Am^2^·kg^–1^) was recalculated to volume magnetization (A·m^–1^) using the density of the BaFe_12_O_19_ crystal phase obtained from the XRD pattern (∼5200 kg·m^–3^). As shown in panels a–c, magnetization of the obtained sample occurs smoothly, without noticeable steps, proving that formation and reversal of the magnetic field are uniform within the material. The sample showed semihard ferromagnetic-type hysteresis with relatively large values of remanent magnetization (*M*_r_) and coercivity (*H*_c_). This is followed by the magnetization saturation (*M*_s_) value, as determined from the fitted law of approach to saturation (LAS),^52^ presented in Figure 7d for the reversible part of the hysteresis in the first quadrant. Based on the fitted LAS function, the effective magnetic anisotropy of the synthesized sample (*K*_eff_) was calculated from parameter *b*, according to the known relation for materials with hexagonal symmetry.^53^
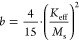
A summary of these values is presented in Table 2. However, all of them fit the expected behavior of BaFe_12_O_19_ compared to, e.g., spinel-type ferrites. Notably, they are generally lower than values reported previously for the high-quality samples.^54^ In the case of the *H*_c_ and *K*_eff_ values, the reason for this could be connected with the synthesized plates being elongated in a direction perpendicular to the easy magnetization axis of the ferrite, resulting in a competition between magnetocrystalline and shape anisotropy of the final material. Therefore, magnetization reversal should require a lower energy barrier than for the bulk structure, effectively lowering values of the determined parameters, especially *K*_eff_ and *H*_c_.^55^ Furthermore, analysis of the *M*(*T*) relation was also performed in the temperature range between 5 and 300 K.

**Table 2:** Summation of the Magnetic Properties Obtained for the Parred BaFe_12_O_19_ Sample

sample	*M*_s_ (Am^2^·kg^–1^)	*H*_c_ (T)	*M*_r_ (Am^2^·kg^–1^)	*K*_eff_ (MJ·m^–3^)	β (K^–3/2^ × 10^–5^)	refs
BaFe_12_O_19_	54	0.08	22	0.104	6.015	in this work
	72	Up to 0.7	n.d.	0.330	4.859–6.210	(23,56−58)

Following the measurement, obtained data were fitted to the Bloch law *M* = *M*_0_·(1 – β·*T*^3/2^) for 75 K ≤ *T* ≤ 300 K, giving parameter β = 6.015 × 10^–5^ K^–3/2^, as shown in Figure 7e. The magnetization plateau for *T* < 75 K would indicate the occurrence of magnetic frustration at this low-temperature range. Noteworthy, the β coefficient is known to be inversely proportional to the exchange interactions, influencing the Curie temperature (*T*_C_) of the material. In this regard, comparing obtained β to other studies shows that its value is similar,^56^ suggesting that no significant differences in the *T*_C_ are expected in the case of the analyzed sample.

Finally, performed analysis of the magnetic properties indicated that the prepared material does not follow its expected bulk structure behavior in a strict way, which might be connected with reduction dimensions along the *c*-axis. Nevertheless, after the magnetization, the sample clearly preserves its magnetization in the absence of an external field (*M*_r_ = 22 Am^2^·kg^–1^), which should allow us to create an in situ magnetic field around its particles and study its effect on the photocatalytic reaction. Moreover, the obtained β value suggests that no significant increase of *T*_C_ occurs with respect to the reported bulk values (∼450 °C) and that simple calcination above this temperature should demagnetize its microscopic structure. In this regard, it was found suitable for the further preparation of the composites.

### 3.3 Characterization of the Magnetic Composite Materials

**Figure 8:**
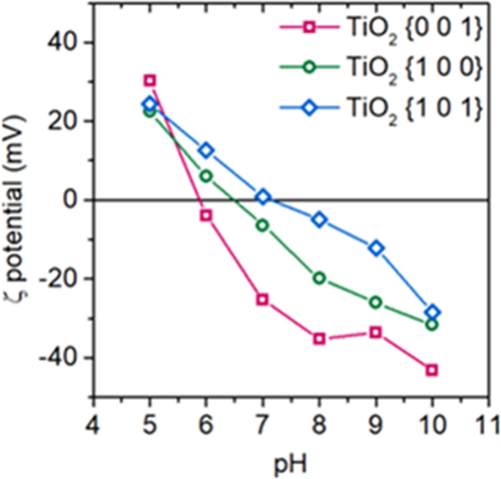
ζ potential analyses for the TiO_2_ samples before combining with BaFe_12_O_19_ as a function of pH and exposed crystal facet.

Prepared BaFe_12_O_19_ samples were combined with three different TiO_2_ morphologies, resulting in composites where TiO_2_ exposed the majority of {0 0 1}, {1 0 0}, or {1 0 1} crystal facets analogically to other recent studies.^11,59,60^ The change of the TiO_2_ surface charge, depending on pH and exposed crystal facet, is presented in Figure 8.

**Figure 9:**
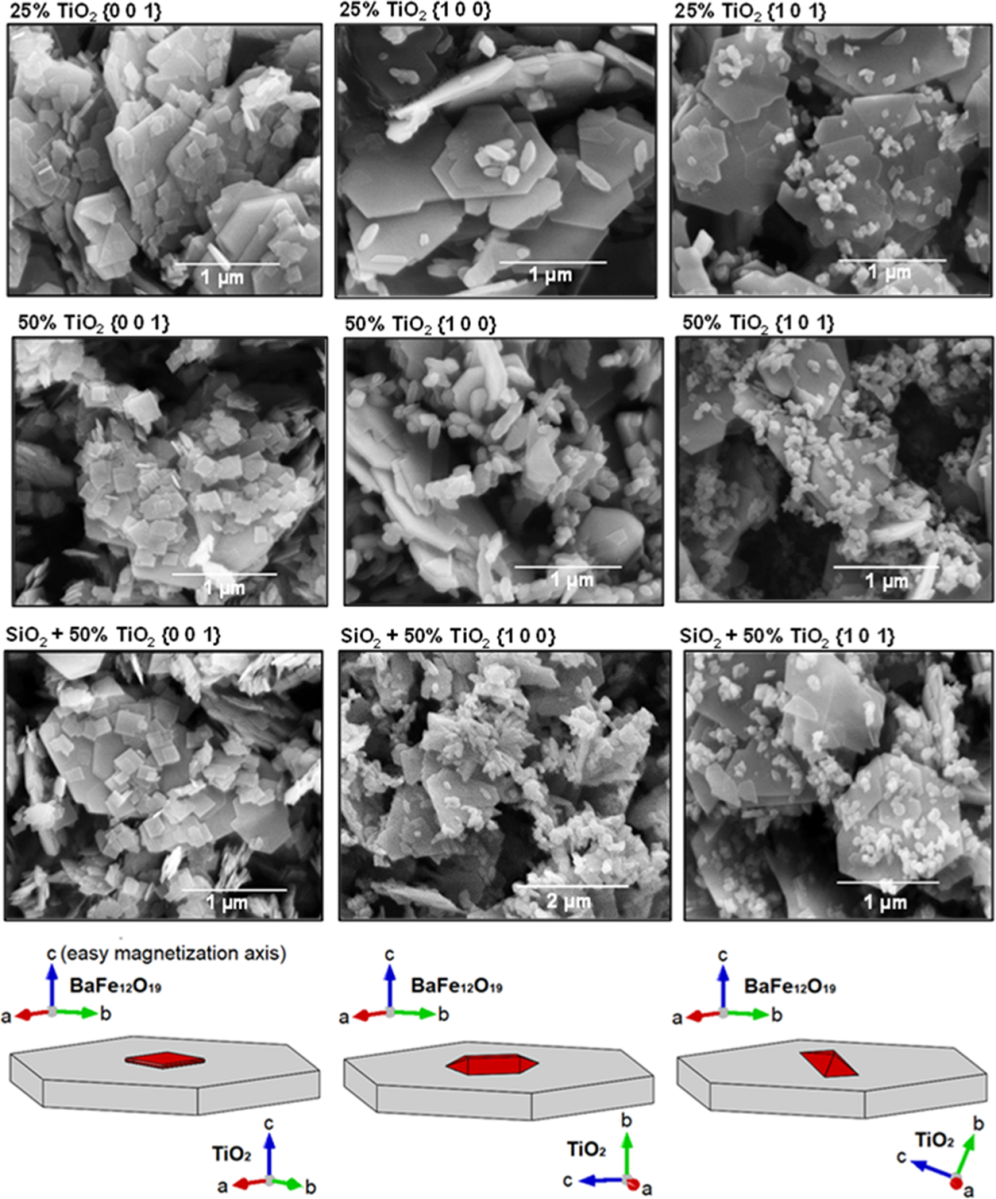
SEM images of the prepared composites, together with the scheme of the idealized orientation between BaFe_12_O_19_ and anatase crystal structures (ferrite as gray plates and TiO_2_ as red particles).

The presence of both phases in the final composite samples with the designed TiO_2_ contents of 25% and 50% (wt) was confirmed by the XRD analysis and SEM observations. In each case, only BaFe_12_O_19_ and anatase TiO_2_ reflections were observed in the XRD patterns, presented in detail in Figures S1 and S2 in the Supporting Information (absence of the α-Fe_2_O_3_ signals results probably from decreasing its amount below the detection limit, as well as reduction of the sample amount taken for analysis, comparing with pure ferrite). As shown in Figure 9, the deposition of the TiO_2_ nanoparticles on the BaFe_12_O_19_ plates is observed, with their morphology depending on the exposed facet. Furthermore, the increased TiO_2_ content resulted in the particles being distributed more densely on the ferrite surface. Noteworthy, this also leads to increased aggregation of TiO_2_, especially noticeable for the {1 0 0} structures. This is a logical consequence of increasing the amount of TiO_2_ with a limited BaFe_12_O_19_ surface left to create the interface. In this regard, composites with higher amounts of TiO_2_ were not investigated in this study, since the limitation of the possible BaFe_12_O_19_/TiO_2_ interface will also limit the effect of their interactions on the final activity of the composite (that is, for high amounts of aggregated TiO_2_, not connected to the ferrite, final properties will start to depend more on the properties of TiO_2_ itself, rather than the TiO_2_/BaFe_12_O_19_ interface).

**Figure 10:**
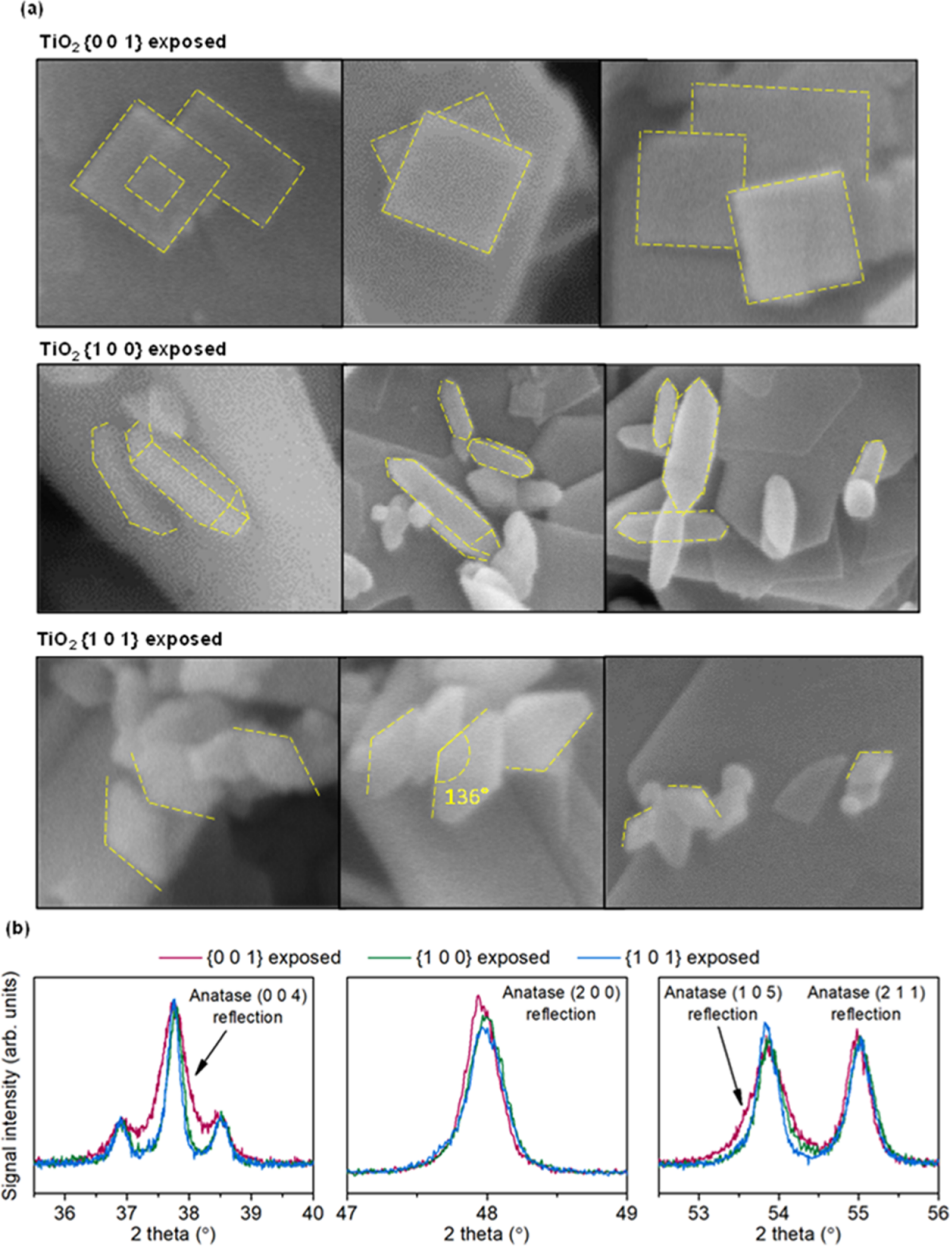
SEM images showing a detailed morphology of the deposited TiO_2_ (a) together with the closeup of the selected XRD reflections of the control TiO_2_ samples (b).

A detailed analysis of the morphology of deposited TiO_2_ is presented in Figure 10a. As observed, rectangular platelets, rods, and octahedrons are formed in each case, which match the expected shape of the nanocrystals. In the case of the {0 0 1} facets, the formation of the 2D plates results from the exposition of the 2 equiv planes (the (0 0 1) and (0 0 1̅)), thermodynamically stabilized by the coadsorption of HF and *n*-butanol during the synthesis.^61^ Additional analysis of the XRD reflections for the control TiO_2_ samples showed that for this nanostructure, selective broadening of the (0 0 4) and (1 0 5) reflections occurs, which is in agreement with the reduction of the crystal structure along the [0 0 1] direction (Figure 10b). In the case of {1 0 0} facets, four sides of the rods correspond to the four equivalent planes that adopt the (1 0 0) structure. Although it might be noticed that in the case of this sample, the symmetry of the particles is occasionally broken, possibly due to the calcination, elongated particles with parallel sides are systematically observed on the surface of the ferrite, which matches preferred growth along the [0 0 1] direction and resulting exposure of the {1 0 0} facets. This is also in accordance with the thermodynamic stabilization of the {1 0 0} planes due to the oxygenation of the surface,^62^ occurring due to the highly basic conditions during the preparation. Finally, in the case of the {1 0 1} facets exposed, octahedral nanocrystals are systematically observed, resulting from the exposition of the 8 equiv {1 0 1} planes. Moreover, the neighboring facets form an angle close to 136.6°, as highlighted in Figure 10a, which is a theoretical value of the angle between the (1 0 1) and (1 0 1̅) planes of the anatase crystal structure. Also, a slightly higher intensity of the (1 0 5) reflection than the (2 1 1) one is noticed in the XRD pattern of this sample, which is a characteristic feature of the {1 0 1} exposition.^63^ In this regard, the successful formation of all of the described nanostructures is concluded.

### 3.4 Photocatalytic Degradation of Phenol

**Figure 11:**
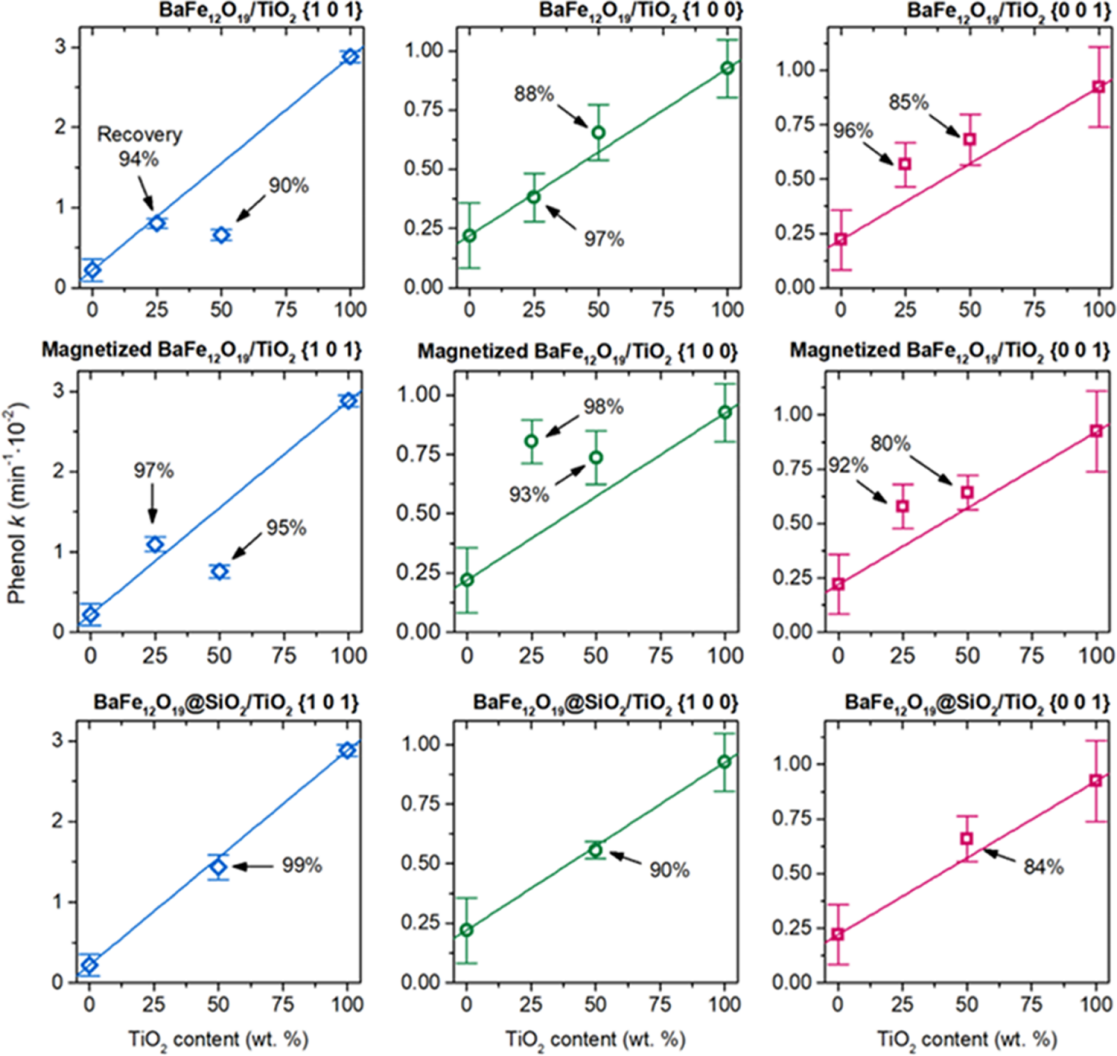
Results of the observed phenol degradation over prepared samples, presented as the calculated rate constant (*k*) of the I-order reaction. The solid lines represent the activity level expected when effectively no interactions are present between both phases.

Ultimately, the prepared magnetic nanocomposites were studied in the reaction with phenol degradation. During these studies, dispersed composites were mixed with a three-dimensional (3D)-printed stirrer mounted to an overhead motor to ensure that no magnetic field was present during the reaction. After each process, composites were separated using a lab magnet, dried, and weighed to calculate the recovery of the material after the process. Figure 11 shows the rate constants of phenol removal, calculated for the first-order reaction, together with the estimated photocatalysts recovery. The fitting of the observed phenol concentration to the first-reaction model is presented in detail in Figure S3 in the Supporting Information. As observed, pure BaFe_12_O_19_ shows relatively low activity, actually being close to the value of spontaneous phenol photolysis under the experimental conditions (0.1 × 10^–2^ min^–1^). On the other hand, the activity of the TiO_2_ structures was at least a few times higher, proving their high phenol degradation efficiency. Noteworthy, the relative activity of the pure TiO_2_ photocatalysts is in agreement with the previous reports on their ability to degrade organic pollutants,^10,20,64^ showing high activity of the {1 0 1} facets. Following the observed rate constants for pure compounds, the performance of the prepared composites can be compared with the results expected from their fraction, as presented in Figure 11 as solid lines. Any deviation from this trend might be seen as the result of interactions between both phases. Interestingly, depending on the system details, different effects might be noticed. For a simple BaFe_12_O_19_/TiO_2_ combination, the activity of the composites changed fairly linearly, with the exception of the 50% TiO_2_{1 0 1} sample, whose activity was significantly reduced. On the other hand, a slightly positive effect can be observed for the composite containing 25% TiO_2_, exposing the majority of the {0 0 1} crystal facet. However, this change is relatively small, and the effect of possible random error cannot be completely ruled out.

Following the results obtained for the composite in direct contact with demagnetized ferrite, further analyses after magnetization of the ferrite or after the introduction of the SiO_2_ layer induce significant changes only in two cases. First of all, the magnetization of the ferrite resulted in more than 2 times higher activity of the 25% TiO_2_{1 0 0} sample compared to the demagnetized state. Noteworthy, the same trend was noticed for the 50% TiO_2_{1 0 0} composite. The higher effect observed for 25% TiO_2_{1 0 0} than 50% TiO_2_{1 0 0} might especially result from better TiO_2_ dispersion on the BaFe_12_O_19_ surface observed in SEM images. Finally, the introduction of the SiO_2_ layer increased the activity of the sample 50% TiO_2_{1 0 1} back to the expected level, while no change was observed for other structures.

Recovered particles were analyzed with XRD to check for the possible change in composition. Qualitatively, no changes were observed for all samples, as shown in Figure S1 in the Supporting Information. Moreover, Table 3 shows the results of the quantitative analysis of the presence of TiO_2_ in all prepared composites. As presented, the prepared composites are generally within ±5% error to their designed composition, which is reasonable due to unavoidable imperfections of the deposition process and measurement errors. Moreover, the composition of the prepared materials is not changed due to the reaction, showing variance mostly within ±2% of the TiO_2_ content. These results confirmed the successful separation of the composites as well as their stability during the reaction. Nevertheless, it can be noted that in the case of the 50% TiO_2_{0 0 1} sample, some loss of the TiO_2_ was observed during the separation process, as shown in Figures S4 and S5 in the Supporting Information. It can result from the large scattering and absorption coefficients of these particles, reported previously.^11^

**Table 3:** Summation of the XRD Quantitative Analysis of the Refined Composite Patterns, Performed before and after the Photocatalysis Process

	TiO_2_{1 0 1} facets			TiO_2_{1 0 0} facets			TiO_2_{0 0 1} facets		
sample	25%	50%	50% SiO_2_	25%	50%	50% SiO_2_	25%	50%	50% SiO_2_
TiO_2_ % before	28.2	51.4	53.4	22.8	44.7	48.5	24.9	52.1	54.0
TiO_2_ % after	27.7	49.9	55.9	23.2	44.9	48.1	23.6	51.9	56.2
χ^2^ before	1.22	1.29	1.39	1.29	1.18	1.48	1.12	1.15	1.21
χ^2^ after	1.18	1.20	1.29	1.19	1.42	1.13	1.06	1.04	1.20

### 3.5 Mechanism Discussion

**Figure 12:**
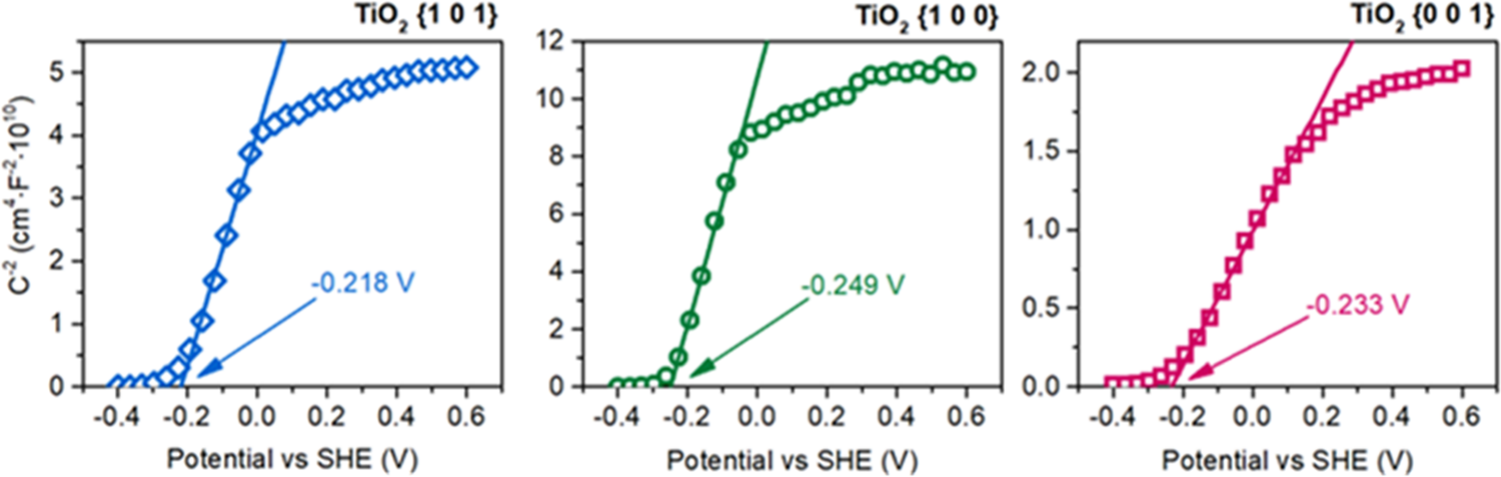
Mott–Schottky plots for the calcined TiO_2_ samples exposing the majority of different crystal facets.

Ultimately, comparing the results obtained for different composite systems (magnetized, demagnetized, and SiO_2_-covered ferrite) enables the discussion of the possible effect of complex interactions between both components concerning different orientations between both phases and possible anisotropy of their properties as well as the effect of the BaFe_12_O_19_ inner magnetic field. Noteworthy, the combination of TiO_2_ with BaFe_12_O_19_ always reduces its activity, which is connected with the negligible activity of BaFe_12_O_19_ itself, resulting from the location of conduction and valence bands (calculated from Mott–Schottky analysis and band gap), as both these values suggest that charge transfer to oxygen and water to form reactive species should be hindered (the potentials for H_2_O/^•^OH and O_2(aq)_/^•^O_2_^–^ are approximately 2.31 and −0.16 V, respectively^65^). However, the exact activity change when both phases are combined starts to depend on the investigated details, proving their influence on the process. First of all, a significant reduction of the {1 0 1} activity when combined with the ferrite can be especially connected to the electron transfer from TiO_2_ to BaFe_12_O_19_. This was further confirmed with the Mott–Schottky analysis of the calcined TiO_2_ samples, as presented in Figure 12. As shown, the potential of the conduction band edge of all TiO_2_ nanostructures is fairly similar and is approximately 0.8 V lower than that for BaFe_12_O_19_, confirming the preferred electron transfer from anatase to ferrite. This process is especially undesired for the nanoparticles exposing {1 0 1} facets, which are known to be reductive in nature, and electrons play a crucial role in their ability to generate reactive oxygen species.^66,67^ Therefore, direct electric contact between inactive BaFe_12_O_19_ and TiO_2_ exposing {1 0 1} facets is especially unfavorable. The introduction of SiO_2_ prevents electron transfer, resulting in higher photocatalytic activity observed for the composite material. This material shows the highest activity within prepared composites, in accordance with {1 0 1} being exceptionally suitable for phenol degradation, as shown before when compared to the TiO_2_ P25 standard.^11^ However, such significantly negative electronic interactions with BaFe_12_O_19_ imply that other composite systems, with maximized {1 0 1} content and minimized electron transfer, might be better suited for practical application. Interestingly, the same effect was not observed for the composites exposing {1 0 0} and {0 0 1} TiO_2_ facets. It might be connected to the fact that these facets are preferentially oxidative, and partial transfer of electrons to the ferrite might not be decisive for their ability to generate reactive species and induce a degradation process.^12,68^

**Figure 13:**
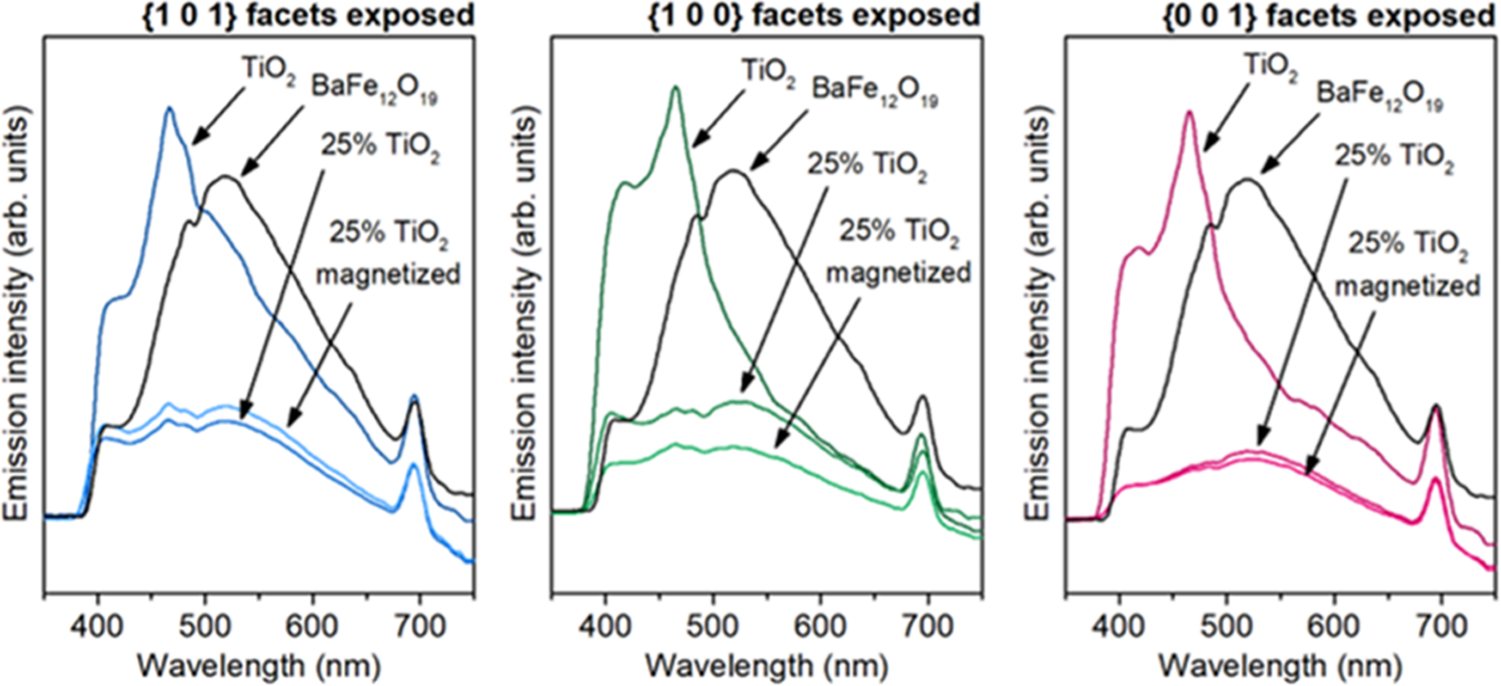
Photoluminescence emission spectra of the pure compounds and 25% TiO_2_ composites in the magnetized and demagnetized states of the ferrite.

Furthermore, the most interesting finding is a significant change of observed activity for the 25% TiO_2_{1 0 0} composite, simply as the result of BaFe_12_O_19_ magnetization. This observation is unique for the TiO_2_ exposing {1 0 0} facets, and the effect is too strong to result from the random error (the activity of this sample is actually comparable to pure TiO_2_, despite its 4 times lower content). The photoluminescence measurements showed that the improved activity of the magnetized composite sample resulted from the effect of the inner magnetic field. As shown in Figure 13, the composite with 25% TiO_2_ exposing {1 0 0} facets showed a significant change in the emission intensity, suggesting that the BaFe_12_O_19_ inner magnetic field suppressed the recombination of charge carriers. Noteworthy, these results showed that interactions between the magnetic field and TiO_2_ are anisotropic and depend on their mutual orientation. However, at this point, it is not possible to distinguish to what extent this anisotropy results from bulk properties (e.g., different mobility of charge carriers in different directions^69^) or from the properties of the exposed facet itself. The obtained results indicate that under the magnetic field, the electronic structure of {1 0 0} is changed toward lower energies, forming more stable states at the surface. Increased stability of such surface states might both reduce the energy of the facet and promote the trapping of long-lived charge carriers at the surface, ultimately leading to reduced recombination and increased activity.

## 4 Conclusions

In the present work, detailed insights into complex electronic and magnetic interactions that can be designed within multicomponent, facet-engineered magnetic photocatalysts are described for the first time. Deposition of faceted TiO_2_ on the surface of single-crystalline BaFe_12_O_19_ microplates allowed for in situ fixing of the orientation between both phases, resulting in different interfaces and different orientations between the faceted TiO_2_ and uniaxial inner magnetic field of the ferrite. Based on the photocatalytic activity analyses, it was noticed that both positive and negative interactions are possible, depending on the system details. Specifically, electron transfer from the {1 0 1} anatase facets to the ferrite phase significantly reduced the final photocatalytic activity. This is in accordance with recent reports showing the importance of the reduction process on the generation of reactive species on {1 0 1} surfaces. For such composites, the introduction of insulating SiO_2_ is especially desired, leading to their high activity when no charge transfer to ferrite occurs. Simultaneously, the analogical electron transfer less influenced the activity of {1 0 0} and {0 0 1} facets, confirming that different effects of the same junction can be expected, depending on the exposed crystal facet. Furthermore, the inner magnetic field of BaFe_12_O_19_ was found to increase the activity of the composites over 2 times for the TiO_2_ sample, exposing {1 0 0} crystal facets. It suggests that the interactions between a magnetic field and TiO_2_ are anisotropic and depend on either their mutual orientation or the electronic structure of the exposed surface. A sharp distinction between bulk and surface effects would require further studies; nevertheless, these results show for the first time that a remarkable activity increase might be achieved due to the action of a magnetic field created in situ within the ferromagnet–photocatalyst composite. However, to observe such an effect, it require a system to be carefully designed. In this regard, further studies in this direction might be desired to help increase the activity of different photocatalytic systems.

## Supporting Information Available

Detailed XRD patterns; results of the Rietveld refinement; observed kinetics of phenol degradation; and snapshots of the performed magnetic separation (PDF)
