# Supplementary material for: Insight into (Electro)magnetic Interactions within Facet-Engineered BaFe12O19/TiO2 Magnetic Photocatalysts
Source: ACS Appl Mater Interfaces. 2023 Nov 22;15(48):56511–25. doi: 10.1021/acsami.3c13380 (PMC10711715; doi:10.1021/acsami.3c13380)
Supplement: Supplementary file 1 — am3c13380_si_001.pdf [file am3c13380_si_001.pdf]

Supporting Information

Insight into (electro)magnetic interactions within facet-engineered $BaFe_{12}O_{19}/TiO_2$ magnetic photocatalysts

Szymon Dudziak[□,*], *Cristina Gómez-Polo*[†], *Jakub Karczewski*[□], *Kostiantyn Nikiforow*[‡] *and*

Anna Zielińska-Jurek[□,*]

[□] Department of Process Engineering and Chemical Technology, Gdansk University of Technology, G. Narutowicza Street 11/12, 80-233, Gdansk, Poland,

[□] Institute of Nanotechnology and Materials Engineering, Gdansk University of Technology, G. Narutowicza Street 11/12, 80-233, Gdansk, Poland

[‡] Institute of Physical Chemistry, Polish Academy of Sciences, Kasprzaka Street 44/52, 01-224, Warsaw, Poland

[†] Institute for Advanced Materials and Mathematics, INAMAT[2], Public University of Navarre, Campus de Arrosadía - 31006 Pamplona, Pamplona, Spain

* Correspondence: dudziakszy@gmail.com, annjurek@pg.edu.pl

3. Results and discussion

3.2. Magnetic and electronic properties of the prepared $BaFe_{12}O_{19}$ and $BaFe_{12}O_{19}@SiO_2$

3.2.1. XRD patterns of the prepared composites and TiO_2 control samples

XRD patterns of the prepared composites are presented in Figure S1. All signals correspond either to the $BaFe_{12}O_{19}$ or anatase TiO_2 crystal structure. See main text for details. A weak

signal observed for sample 50% {0 0 1} after process results from its small amount during the analysis.

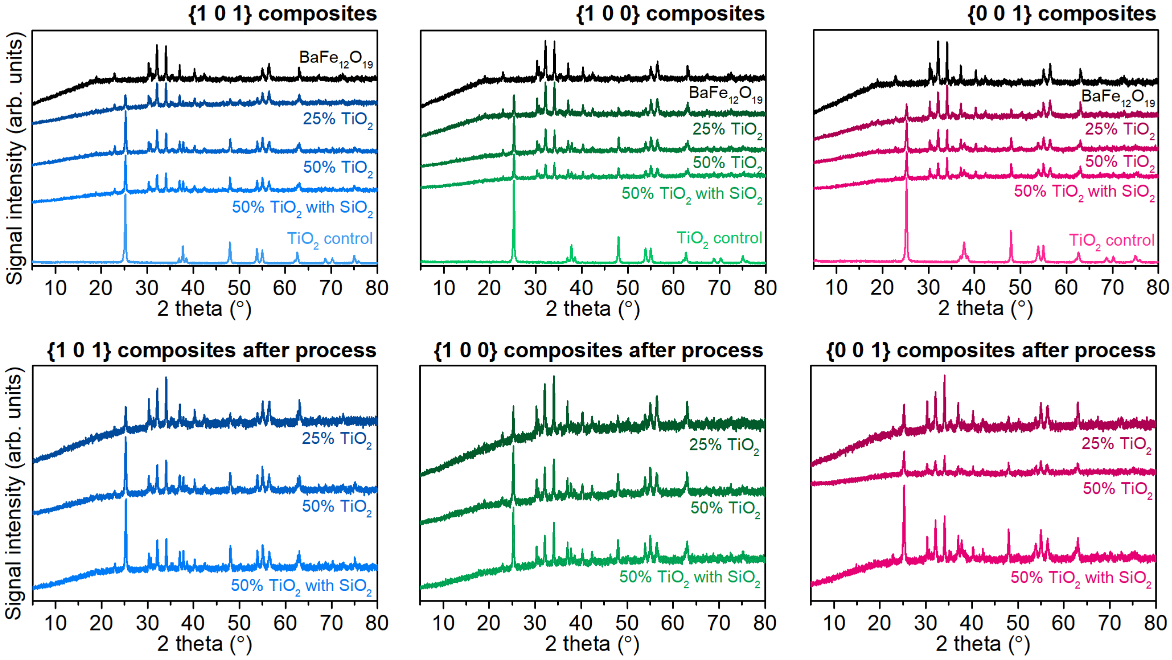

Figure S1. XRD patterns for the prepared composites before and after the photocatalytic reactions. Additional patterns obtained for $BaFe_{12}O_{19}$ and control TiO_2 samples are presented for comparison.

Furthermore, Rietveld refined patterns of the obtained composites are shown in detail in Figure S2 below.

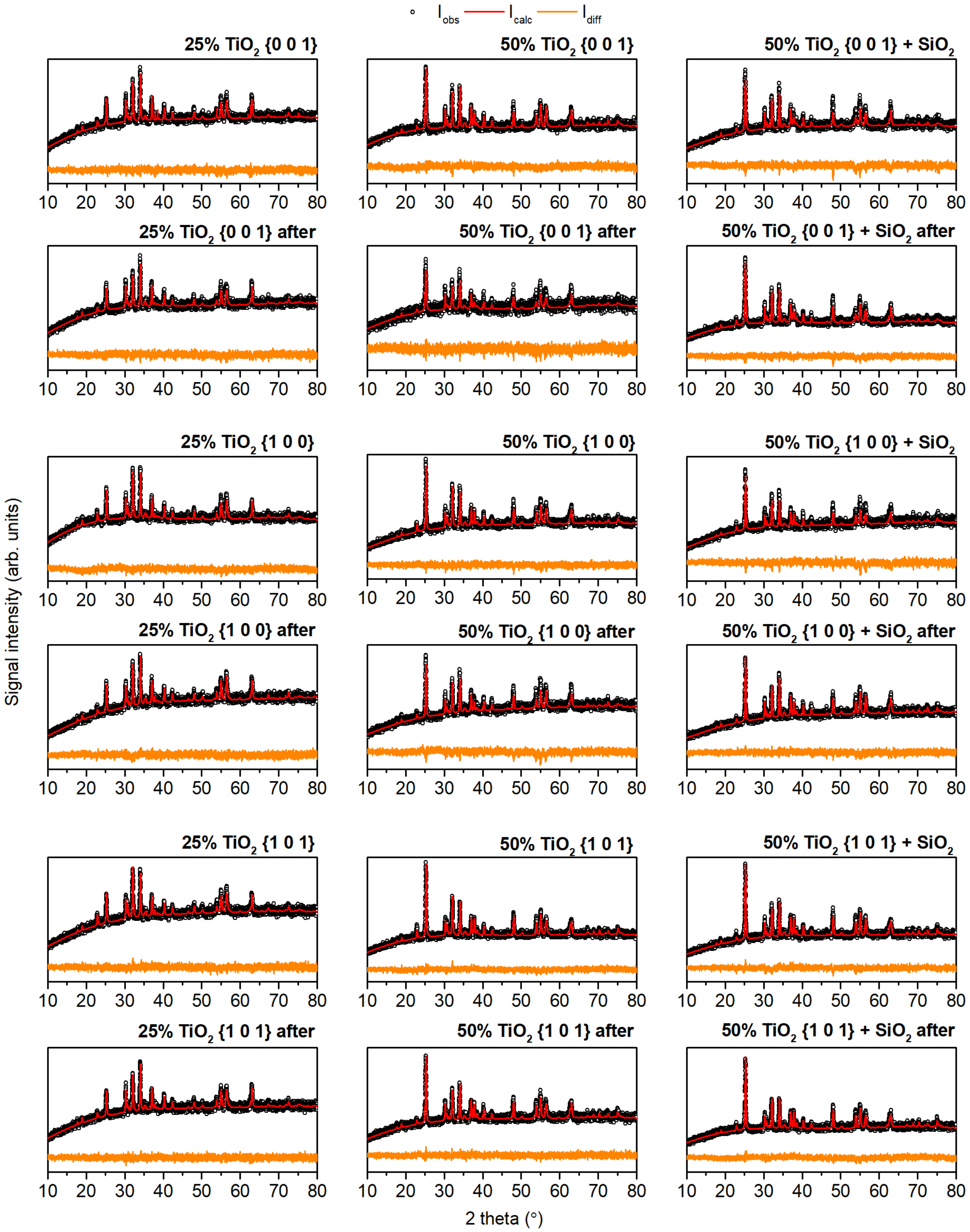

Figure S2. Rietveld refined patterns of the obtained composites before and after the photocatalytic process.

3.4. *Photocatalytic degradation of phenol*

The kinetics of phenol degradation are presented in Figure S3, assuming I-order reaction in each case. Based on the performed fitting, measurement error was estimated and presented in the main text.

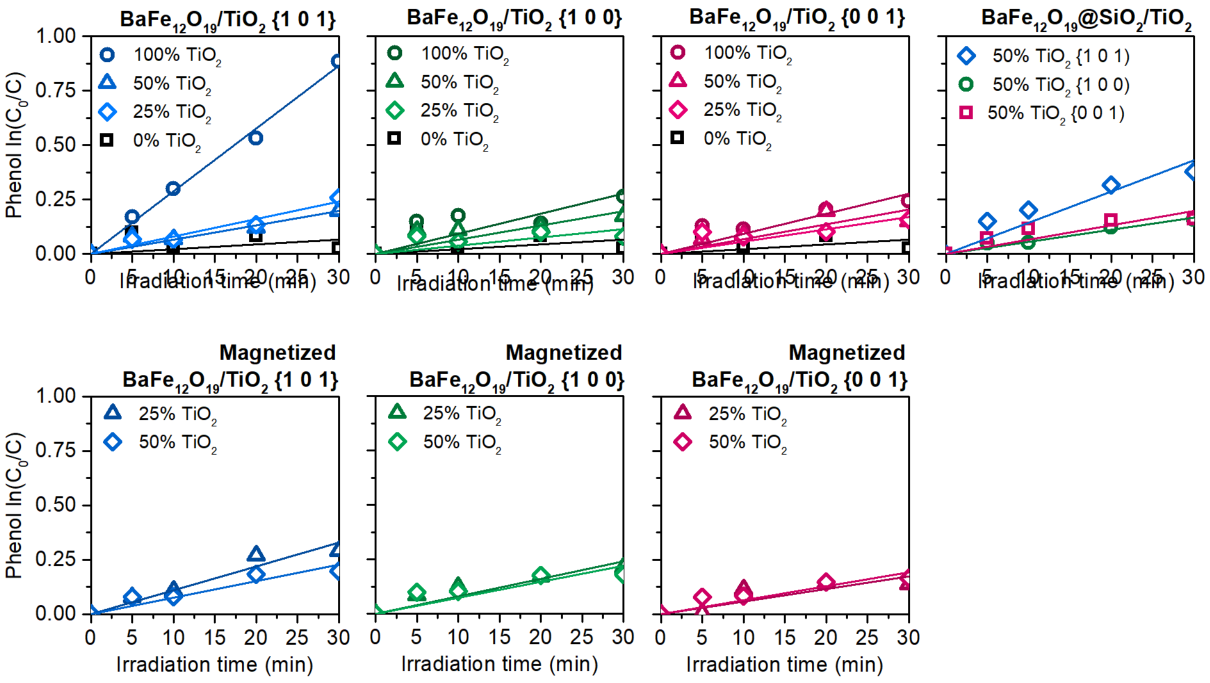

Figure S3. Kinetics of observed phenol degradation over prepared samples. Lines are fitted assuming I-order reaction model. Obtained slopes correspond to the rate constants presented in the main text.

3.5. *Recovery in a magnetic field*

All samples show ability to recover the material inside external magnetic field. In each case, suspension was transferred from the photocatalytic reactor to a weighted beaker, separated using magnet (remaining liquid is discarded), dried and weighted again to obtain efficiency of the recovery. Calculated efficiencies are presented in the main text. Additional snapshots of the separation process recorded for the sample 25% TiO_2 exposing {1 0 0} facets are presented in Figure S4. Full recorded process is available as the separated file.

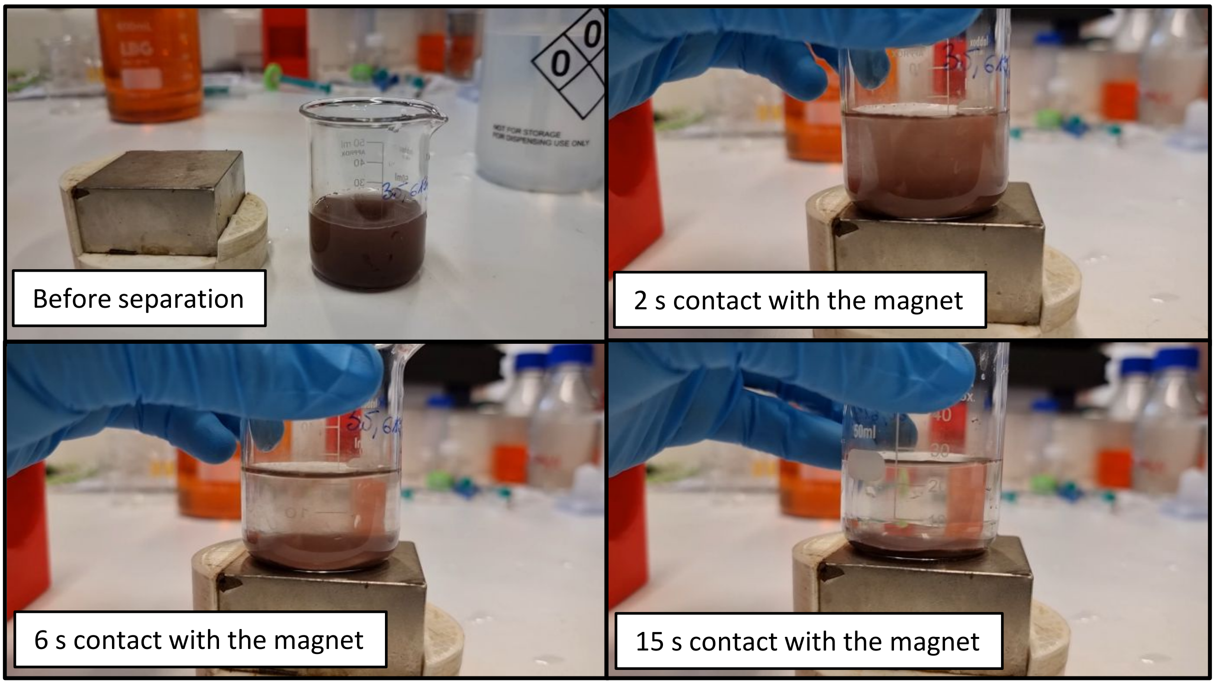

Figure S4. Snapshots of the recorded magnetic separation of the exemplary composite (50% TiO_2 exposing {1 0 0} facets).

Similar separation was observed for all of the prepared samples, with exception of the 50% TiO_2 exposing {0 0 1} facets, where visible amount of TiO_2 was left in the suspension, as presented in Figure S5. This is in agreement with the observed morphology of the composites, presented in the main text.

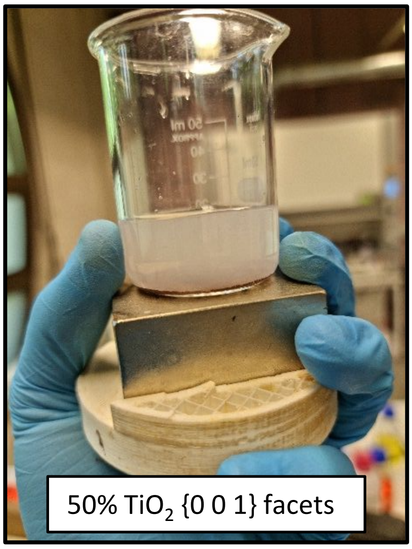

Figure S5. Image of the free TiO_2 particles observed after the separation process of the 50% TiO_2 {0 0 1} composite.
